# The unfolded protein response is activated in disease-affected brain regions in progressive supranuclear palsy and Alzheimer’s disease

**DOI:** 10.1186/2051-5960-1-31

**Published:** 2013-07-06

**Authors:** Lauren D Stutzbach, Sharon X Xie, Adam C Naj, Roger Albin, Sid Gilman, Virginia M Y Lee, John Q Trojanowski, Bernie Devlin, Gerard D Schellenberg

**Affiliations:** 1 Department of Pathology and Laboratory Medicine, 3630 Hamilton Walk. Perelman School of Medicine, University of Pennsylvania, Philadelphia, PA 19104, USA; 2 Center for Neurodegenerative Disease Research, 3 Maloney Building, 3600 Spruce St Philadelphia, PA 19104, USA; 3 Center for Clinical Epidemiology & Biostatistics, University of Pennsylvania, 618 Blockley Hall, 423 Guardian Dr, Philadelphia, PA 19104, USA; 4 Department of Neurology, University of Michigan, 1500 E. Medical Center Dr, Ann Arbor, MI 48109, USA; 5 VAAAHS GRECC, 2215 Fuller Rd, Ann Arbor, MI 48109, USA; 6 Michigan Alzheimer disease Center, 2101 Commonwealth Blvd, Ann Arbor, MI 41805, USA; 7 Department of Psychiatry, University of Pittsburgh School of Medicine, Pittsburgh, PA 15260, USA; 8 607 Stellar Chance Laboratories, 422 Curie Blvd, Philadelphia, PA 19104, USA

**Keywords:** Progressive supranuclear palsy, PERK, Unfolded protein response, EIF2AK3, Alzheimer’s disease

## Abstract

**Background:**

Progressive supranuclear palsy (PSP) is a neurodegenerative disorder pathologically characterized by intracellular tangles of hyperphosphorylated tau protein distributed throughout the neocortex, basal ganglia, and brainstem. A genome-wide association study identified *EIF2AK3* as a risk factor for PSP. *EIF2AK3* encodes PERK, part of the endoplasmic reticulum’s (ER) unfolded protein response (UPR). PERK is an ER membrane protein that senses unfolded protein accumulation within the ER lumen. Recently, several groups noted UPR activation in Alzheimer’s disease (AD), Parkinson’s disease (PD), amyotrophic lateral sclerosis, multiple system atrophy, and in the hippocampus and substantia nigra of PSP subjects. Here, we evaluate UPR *PERK* activation in the pons, medulla, midbrain, hippocampus, frontal cortex and cerebellum in subjects with PSP, AD, and in normal controls.

**Results:**

We found UPR activation primarily in disease-affected brain regions in both disorders. In PSP, the UPR was primarily activated in the pons and medulla and to a much lesser extent in the hippocampus. In AD, the UPR was extensively activated in the hippocampus. We also observed UPR activation in the hippocampus of some elderly normal controls, severity of which positively correlated with both age and tau pathology but not with Aβ plaque burden. Finally, we evaluated *EIF2AK3* coding variants that influence PERK activation. We show that a haplotype associated with increased PERK activation is genetically associated with increased PSP risk.

**Conclusions:**

The UPR is activated in disease affected regions in PSP and the genetic evidence shows that this activation increases risk for PSP and is not a protective response.

## Background

Progressive supranuclear palsy (PSP) is a late-onset neurodegenerative movement disorder clinically characterized by vertical gaze palsy, poor balance and frequent falls, as well as cognitive impairment and dementia [1,2]. The primary symptoms of PSP are consistent with the observed neuropathology, mainly neuronal degeneration in the brainstem, particularly the pons and medulla [3]. Postmortem pathological analysis of these brain regions in PSP patients reveals numerous intracellular neurofibrillary and glial tangles comprised of hyperphosphorylated protein tau (htau). Thus PSP, along with Alzheimer’s disease (AD), belongs to a group of disorders collectively known as tauopathies, as all these disorders show abundant tau aggregates or inclusions as prominent neuropathologic features. Other tauopathies include frontotemporal dementia with Parkinsonism linked to chromosome 17 (FTDP-17), corticobasal degeneration (CBD), and Pick’s disease [4]. Some mutations in the gene *MAPT*, which encodes tau, can result in a PSP phenotype [5-9], while common variants in the *MAPT* region are associated with PSP susceptibility [10-13]. Thus, genetic studies as well as our data here indicate that tau is clearly linked to PSP pathogenesis.

Schellenberg and colleagues recently completed a genome-wide association study (GWAS) for PSP risk loci [10]. One of the genes identified was eukaryotic translation initiation factor 2 alpha kinase 3 (*EIF2AK3*), which encodes the protein pancreatic endoplasmic reticulum kinase (PERK). PERK is an endoplasmic reticulum (ER) membrane protein that acts as a stress sensor in the ER unfolded protein response (UPR). In addition to PERK, there are two other stress sensors (both of which are also ER membrane proteins): inositol-requiring enzyme 1α (IRE1α) and activating transcription factor 6 (ATF6; [14]).

All three arms of the UPR activate when the chaperone immunoglobulin binding protein (BiP), normally bound on the luminal side of each protein, dissociates in order to aid in the folding of accumulated unfolded proteins in the ER lumen. Dissociation of BiP from PERK and IRE1α facilitates their activation by promoting homodimerization and *trans*-autophosphorylation [15]. ATF6 is then activated *via* a cleavage event and subsequently translocated from the ER to the nucleus [16]. Each of the three branches of the UPR initiates discrete signaling cascades in response to the accumulation of unfolded proteins in the ER lumen. The role of the UPR is to restore protein homeostasis by upregulating chaperone production [17,18], attenuating translation, promoting degradation of misfolded proteins *via* ER-associated degradation (ERAD; [19], and promoting autophagy [20]. Prolonged ER stress can trigger apoptosis [14,21].

The PERK arm of the UPR acts primarily on translation. When PERK is activated (thus becoming phosphorylated PERK, or pPERK), a kinase domain on the cytosolic side of the protein phosphorylates eukaryotic translation initiation factor 2α (eIF2α or peIF2α when phosphorylated). peIF2α is a less active form of the protein, and its decreased efficiency slows general translation initiation and promotes translation of activating transcription factor 4 (ATF4). ATF4 promotes transcription of genes that enhance amino acid uptake and protect against oxidative stress [22]. Elements of the PERK pathway are also involved in regulating autophagy, a process that degrades misfolded proteins [23,24]. Thus, genetic variation that results either in alteration of PERK protein function or significant changes in the amount of PERK would perturb several crucial stress-response pathways.

Several neurodegenerative disorders, including PSP, are characterized by pathological aggregates of misfolded proteins in the brain. Previous work showed that the UPR is activated in post-mortem AD brains [25], as well as in the brains of patients with frontotemporal lobar degeneration with tau inclusions (FTLD-tau) [26], PD [27], amyotrophic lateral sclerosis (ALS) [28,29], and multiple system atrophy (MSA) [30]. Nijholt *et al.* (2011) reported evidence of UPR activation in the hippocampus and, to a lesser extent, the *locus coeruleus* and putamen of PSP patients.

We investigated activation of PERK and eIF2α in postmortem brains from subjects with PSP and AD, as well as from normal elderly subjects. We used antibodies that recognize the phosphorylated species of PERK and eIF2α, *i.e.* the activated forms of these 2 proteins (pPERK and peIF2α, respectively). Our primary goal was to investigate the brain regions most affected by tau pathology in PSP. We searched for evidence of PERK and eIF2α activation in the pons, medulla and midbrain, regions affected in PSP, in the hippocampus and frontal cortex, which are regions affected in AD, and in the cerebellum, a brain region which is relatively spared in both diseases, although the deep cerebellar nuclei and cerebellar cortex may harbor modest amounts of tangles and plaques, in PSP and AD, respectively. We also looked at PERK and eIF2α activation in young controls to determine if ER stress is activated in normal aging. Our results indicated that PERK and eIF2α activation pllels the pattern of neuropathology in PSP and AD. In normal hippocampus, activation increases with age and correlates with tau but not Aβ amyloid pathology. We also examined coding haplotypes that were previously shown to affect PERK activation [31]. We found that the haplotype that corresponding to the highest PERK activation is in linkage disequilibrium (LD) with the high risk allele of the top PSP GWAS marker, indicating that UPR activation increases PSP risk and is not a protective response in PSP.

## Results

### The PERK arm of the UPR is activated in PSP

To determine whether the UPR is activated in PSP, we stained post-mortem human brain tissue from PSP and AD patients as well as normal elderly controls using antibodies against pPERK and peIF2α, the activated forms of these proteins. We chose six brain areas to stain for PERK and eIF2α activation: the pons, medulla, and midbrain (affected in PSP), the hippocampus and frontal cortex (affected in AD), and the cerebellum, which is relatively spared in both diseases.

**Figure 1:**
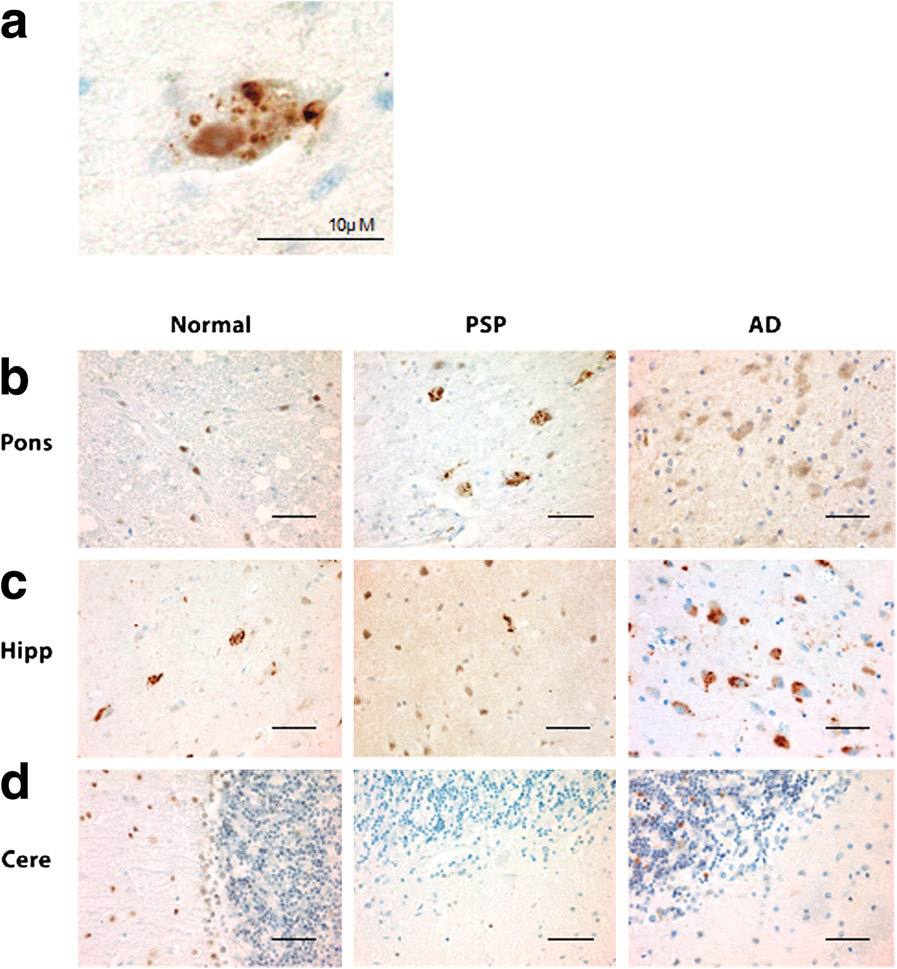
**pPERK is activated in PSP, AD, and normal brain. a.** Example of a cell with pPERK immunoreactive puncta in the pons of a PSP case. **b-d.** Representative fields showing pPERK staining of pons, hippocampus, and cerebellum in normal, PSP, and AD cases. Scale bars are 50 μm unless otherwise indicated.

**Figure 2:**
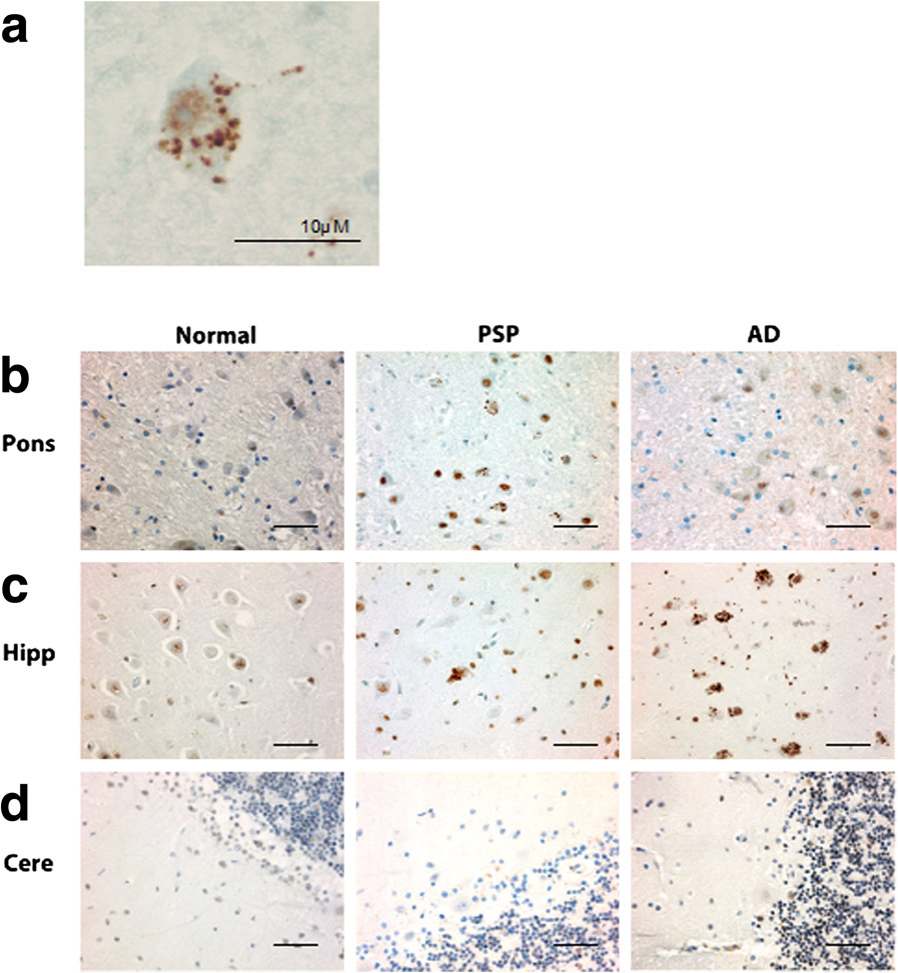
**peIF2α is activated in PSP, AD, and normal brain. a.** Example of a cell with peIF2α puncta in the pons of a PSP case. **b-d.** Representative fields from peIF2α staining of pons, hippocampus, and cerebellum in normal, PSP and AD cases. Scale bars are 50 μm unless otherwise indicated.

**Figure 3:**
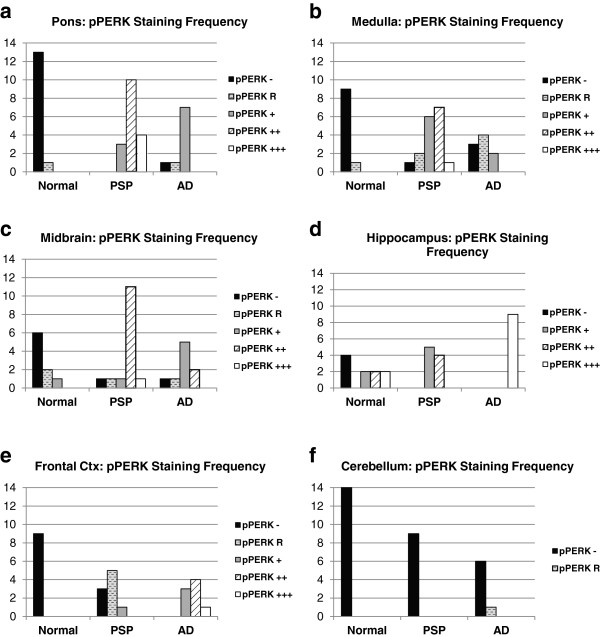
**Frequency of pPERK staining scores in PSP, AD, and normal brain.** Distribution of pPERK staining scores. +++ = widespread activation, ++ = moderate activation, + = diffuse activation, R = rare activation, - = no activation. Y-axis indicates number of cases with a particular pPERK staining score. All P-values obtained using Fisher exact test. **a-c.** PSP cases had the strongest pPERK staining in the pons (PSP vs. Normal: p = 3.8E-9) and the medulla (PSP vs. Normal: p = 6.1E-7), as well as moderate staining in the midbrain (PSP vs. Normal: p = 6.0E-6) which was affected in all PSP cases. **d-e.** AD cases had the strongest pPERK staining in the hippocampus (AD vs. Normal: p = 0.0006) and moderate staining in the frontal cortex (AD vs. Normal: p = 4.1E-5) both of which were affected in AD. **f.** No cases had significant pPERK staining in the cerebellum.

**Figure 4:**
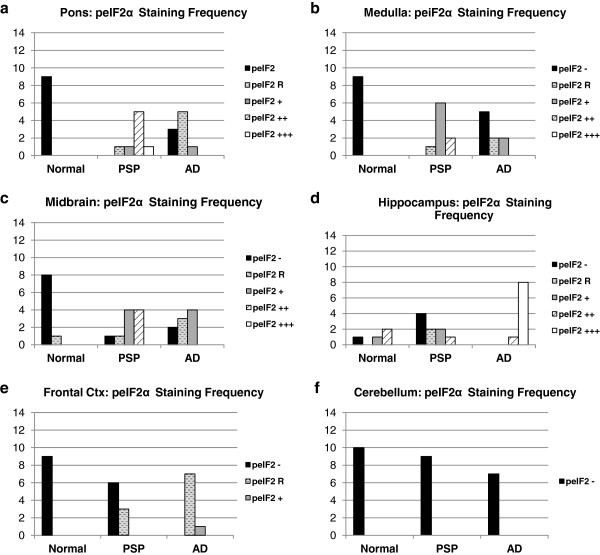
**Frequency of peIF2α staining scores in PSP, AD, and normal brain.** Distribution of peIF2α staining scores. +++ = widespread activation, ++ = moderate activation, + = diffuse activation, R = rare activation, - = no activation. Y-axis indicates number of cases with a particular peIF2α staining score. All P-values obtained using Fisher exact test. **a-c.** PSP cases had the strongest peIF2α staining in the pons (PSP vs. Normal: p = 4.1E-5) and the medulla (PSP vs. Normal: p = 6.0E-6), as well as moderate staining in the midbrain (PSP vs. Normal: p = 0.00041) which was affected in all PSP cases. **d-e.** AD cases had the strongest peIF2α staining in the hippocampus (AD vs. Normal: p = 0.0042) and moderate staining in the frontal cortex (AD vs. Normal: p = 4.1E-5) both of which were affected in AD. **f.** No cases had significant peIF2α staining in the cerebellum.

In PSP cases, of the regions tested, the pons, medulla, and midbrain demonstrated the highest degree of pPERK and peIF2α staining (Figures 1b, 2b, 3a-c, and 4a-c) as measured by number of cells showing staining per field (Additional file 1: Figure S1). These are the brain areas most affected by tau pathology in PSP. pPERK and peIF2α staining was punctate and cytoplasmic with some non-specific nuclear staining (Figures 1a and 2a), a pattern observed by others in AD and PD [25,27]. In the pons, all PSP cases showed some cells positive for both pPERK and peIF2α. pPERK was observed in the medulla and midbrain in all but one case for each region. For peIF2α, all cases showed positive cells in the medulla and all but one case showed positive cells in the midbrain.

PSP cases as a group showed significantly more pPERK and peIF2α staining in the pons, medulla, and midbrain compared to elderly controls. For pPERK, only one control subject (age 63) showed “rare” positive cells in the pons and medulla. This is not the same control subject that displayed Lewy body pathology in the medulla. In the midbrain, very few controls were positive for pPERK. For peIF2α, most controls were negative in these brain areas except for a single subject with rare positive cells in the medulla. For AD, there were more positive cases with a higher density of positive cells compared to controls but less than found in PSP (Figures 3 and 4a-c, Table 1).

**Table 1:** P-values for comparison of pPERK and pEIF2α immunoreactivity in PSP, AD, and normal controls for different brain regions

**Comparison groups**		**Pons**		**Medulla**		**Midbrain**		**Hippocampus**		**Frontal cortex**		**Cerebellum**
	**pPERK**	**peIF2α**	**pPERK**	**peIF2α**	**pPERK**	**peIF2α**	**pPERK**	**peIF2α**	**pPERK**	**peIF2α**	**pPERK**	**peIF2α**
**AD vs. Normal**	3.4E-5	0.0045	0.034	0.041	0.0026	0.0037	0.0006	0.0042	4.1E-5	4.1E-5	0.4	1
**PSP vs. AD**	3.8E-4	0.00049	1.7E-4	0.028	4.0E-7	0.009	0.000021	8.2E-5	1.6E-4	0.0049	0.5	1
**PSP vs. Normal**	3.8E-9	4.1E-5	6.1E-7	6.0E-6	6.0E-6	1.4E-4	0.0034	0.073	0.0045	0.1	1	1

P-values are from a Fisher exact test. “E” indicates “x 10^”. pPERK and peIF2α staining in PSP brainstem areas (pons, medulla, midbrain) were significantly greater than in AD or normal brainstem areas. AD brainstem areas had significantly more pPERK and peIF2α staining than normal brainstem areas. In contrast, primary AD-affected brain areas (hippocampus, frontal cortex) had significantly more pPERK and peIF2α staining than PSP or normal hippocampus and frontal cortex. There was no difference in staining between AD, PSP, or normal brains in the cerebellum.

In the hippocampus and frontal cortex, AD cases as a group scored significantly higher than PSP or normal elderly controls for both pPERK and peIF2α staining (Table 1). pPERK and peIF2α staining was especially strong in the AD hippocampus, with nearly all cases demonstrating high levels of positive cells. All PSP cases had mild to moderate pPERK staining in the hippocampus, though not all cases demonstrated peIF2α staining. Surprisingly, many normal elderly controls demonstrated at least a mild level of pPERK and peIF2α positive cells in the hippocampus (Figures 1c, 2c, 3d, and 4d). Staining was generally milder in the frontal cortex than in the hippocampus, although AD cases still scored significantly higher than PSP cases or normal controls (Figures 3e and 4e). PSP cases scored significantly higher than normal controls for pPERK staining but not for peIF2α staining (Table 1). Notably, the pons, medulla, and midbrain are severely affected in PSP [2] but only moderately or mildly affected in AD [32]. Conversely, the hippocampus and frontal cortex are strongly affected in AD [32], but only mildly affected or unaffected in PSP. Thus, PERK activation is strongest in areas of the brain highly affected by pathology in PSP and AD. Nearly all cases were negative for pPERK and peIF2α in the folia of the cerebellum (Figures 1d, 2d, 3f, and 4f), although one AD case showed rare staining in this area, but, in general, there is little to no pathology in this area in PSP or AD, and thus our findings are consistent with the inference that pathology and PERK activation occur in the same disease-affected brain areas.

### Activation of pPERK in htau positive cells

**Figure 5:**
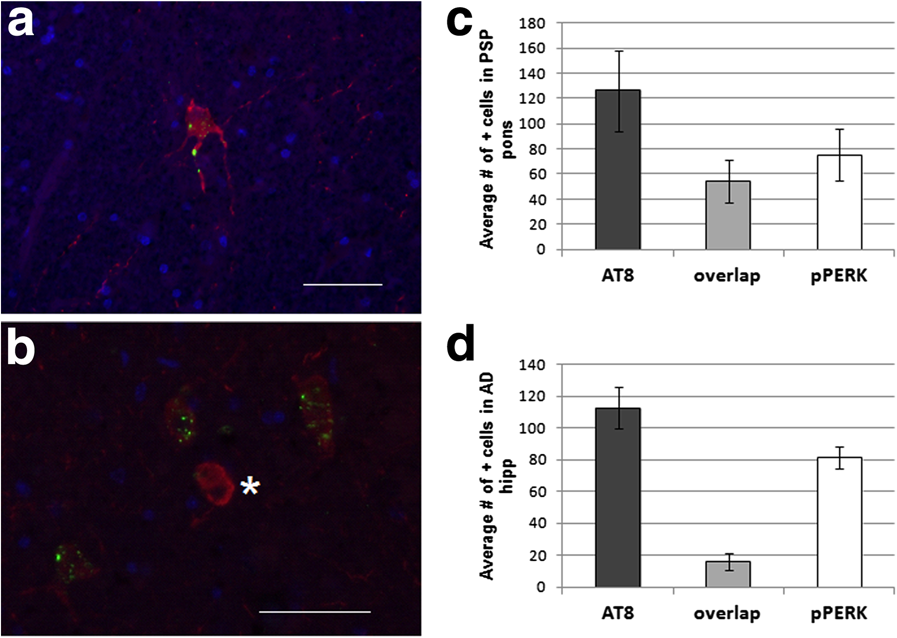
**Hyperphosphorylated tau and pPERK partially co-localize in PSP pons and AD hippocampus. a.** Example of a neuron co-stained for htau (red) and pPERK (green). Tau staining is widespread and diffuse. pPERK staining is punctate and localized to the soma and proximal neurites. **b.** pPERK staining occurred mostly in cells with diffuse, non-fibrillar htau staining. Cells with dense, fibrillar htau staining did not stain for activated PERK (*). **c.** In PSP pons, most pPERK positive cells also stained for htau (72%), whereas fewer than half of htau stained cells (43%) also stained positive for pPERK. **d.** htau and pPERK staining overlapped very little in AD hippocampus (14% and 20%). Scale bars are 50 μm unless otherwise indicated.

We were interested in whether the UPR is activated in cells affected by tau pathology. We performed double immunofluorescence staining for pPERK and htau on sections of pons and hippocampus in PSP, AD, and normal controls (Figure 5a). In PSP pons, an average of 72% of pPERK positive cells were also positive for htau. However, only 43% of htau positive cells were also positive for pPERK (Figure 3c). This substantial overlap is in contrast to AD hippocampus, in which only 20% of pPERK positive cells also stained for htau and only 12% of htau positive cells stained for pPERK (Figure 3d). Overlap between htau and pPERK staining was also low in PSP and normal hippocampus (data not shown). In the pons, overlap between pPERK puncta and htau occurred mostly in cells with diffuse, cytoplasmic htau staining rather than dense, fibrillar staining (Figure 3b). This suggests that PERK is activated in pre-tangle neurons. Hoozemans *et al.*[25] described similar distribution of htau/pPERK staining in AD hippocampus.

### PERK is activated in normal hippocampus

**Figure 6:**
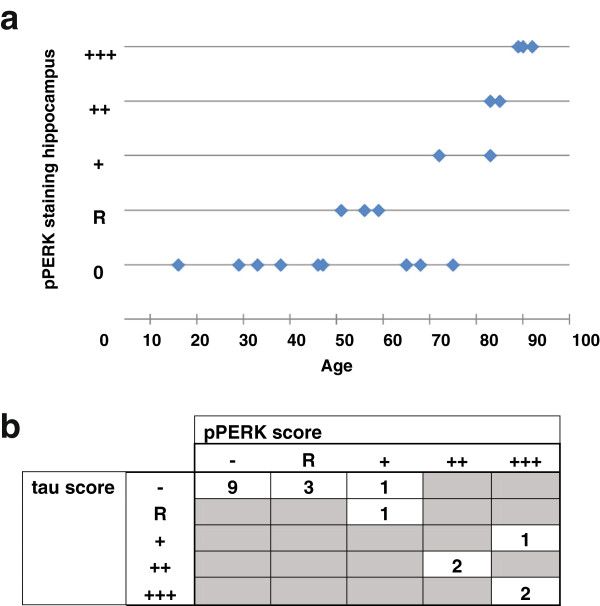
**Severity of PERK activation in normal hippocampus correlates with age and tau pathology. a.** Plot of pPERK staining score (X-axis) versus subject age at death (Y-axis). Each diamond represents one normal subject. Some individuals both young and old were negative for pPERK staining. Of those that stained positive for pPERK (including those showing rare through +++ levels of immunoreactivity), older individuals tended to have more severe pPERK staining scores. **b.** Frequency table plotting htau score against pPERK staining score in normal hippocampus. Htau score and pPERK score were positively correlated (Spearman R: .7523; p = 0.0002). The higher the htau score of an individual hippocampus, the higher the pPERK staining score tended to be. Htau scores were obtained from the CNDR Integrated Neurodegenerative Disease Database [33] using antibody PHF-1.

Une xpectedly, we found pPERK and peIF2α staining in the hippocampus of age-matched elderly normal controls as described above. To follow up on this finding, we expanded our initial control hippocampus sample to include cases with a wide range of ages (range: 16–92, mean: 52.4; see Additional file 1: Table S1). We found that age significantly correlated with the pPERK staining score (Figure 6a). The older the subject, the more likely they were to have high levels of PERK activation in the hippocampus. However, not all aged normal controls demonstrated hippocampal pPERK activation although some subjects at all ages examined here were negative for pPERK staining.

We also found that the level of tau pathology correlated with pPERK staining. The more tau pathology (as measured by PHF-1 staining) in a normal hippocampus, the more likely that the hippocampus was also positive for activated PERK (Figure 6b). All controls negative for pPERK staining were also negative for htau staining; cases with severe pPERK staining scores also scored high for htau. This correlation was significant (Spearman R: 0.7523, p = .0002). There was no correlation between pPERK staining in the hippocampus and Aβ amyloid plaque pathology (as measured by Thioflavin S staining to detect senile plaques); all normals with high pPERK scores and relatively high tau scores in the hippocampus were negative for Aβ amyloid plaques and Lewy bodies (data not shown).

### PERK protein coding variants are associated with PSP risk

Alleles at rs7571971 are significantly associated with PSP risk [10]. To identify other SNPs in high linkage disequilibrium with rs7571971, we evaluated 1000 Genomes data for subjects of European ancestry. As assessed by LD measure r^2^[34], 14 SNPs were in high LD with rs7571971 (r^2^ > 0.8), including the 3 non-synonymous coding variants. Of these 14, none fell in the coding region of any gene besides *EIF2AK3* and all but 5 fell within *EIF2AK3* (Table 2).

**Table 2:** SNPs in high LD with rs7571971

**SNP**	**Gene**	**Distance in base pairs from GWAS hit SNP**	**RSquared**	**DPrime**	**Coordinate_HG18**
rs1805165	EIF2AK3	20460	0.889	1	88656006
rs6739095	EIF2AK3	15287	0.886	0.96	88661179
rs11898161	EIF2AK3	13703	0.925	1	88662763
rs1913671	EIF2AK3	4532	0.886	0.96	88680998
rs11681299	EIF2AK3	6381	0.889	1	88682847
rs867529	EIF2AK3	17922	0.889	1	88694388
rs6731022	EIF2AK3	21684	0.886	0.96	88698150
rs11684404	EIF2AK3	29271	0.886	0.96	88705737
rs11680549	EIF2AK3	30997	0.813	0.957	88707463
rs6547787		34385	0.886	0.96	88710851
rs1606803		37965	0.889	1	88714431
rs62157778		38739	0.889	1	88715205
rs13003510		46139	0.925	1	88722605
rs13001657		51260	0.888	0.96	88727726

The 3 non-synonymous coding variants in *EIF2AK3* were Ser136Cys, Arg166Gln, and Ser704Ala. When haplotypes were constructed from 1000 Genome data, there were two common haplotypes (Table 3): Ser-Arg-Ser (haplotype A) and Cys-Gln-Ala (haplotype B) with predicted frequencies of 0.64 and 0.29, respectively; one uncommon haplotype, Ser-Gln-Ser (haplotype D), with a frequency of 0.06; and 4 rare haplotypes of frequency close to 1/1000. The top PSP GWAS SNP for this gene is rs7571971, a 2-allele polymorphism in *EIF2AK3* intron 2 with a minor allele frequency of 0.25-0.28 [10]. From the 1000 genome analysis, the minor allele for rs7571971 is almost perfectly correlated with haplotype B and the major allele with haplotypes A and D.

**Table 3:** PERK haplotypes

	**rs867529-rs13045-rs1805165**	**Affected amino acids**	**Alleles (%)**	
			**PSP (*n*** **= 994)**	
**Haplotype A**	GCA	Ser136-Arg166-Ser704	1233	(62.5)
**Haplotype B**	CTC	Cys136-Gln166-Ala704	626	(31.7)
**Haplotype D**	GTA	Ser136-Gln166-Ser704	113	(5.7)

**Figure 7:**
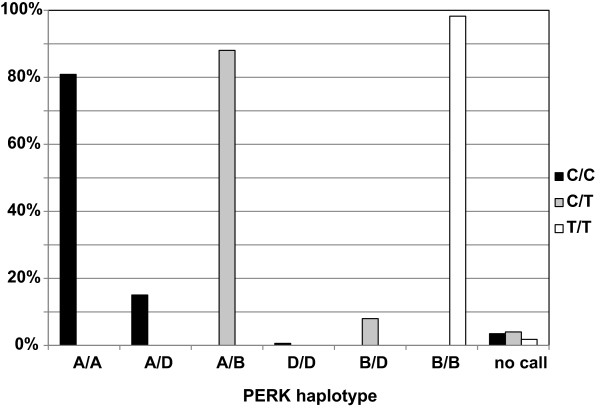
**Comparison of PERK haplotype with GWAS risk allele.** A GWAS for PSP identified a risk locus on chromosome 2 (rs7571971). The common, low risk allele at this locus is cytosine (C) and the PSP risk allele is thymine (T) [10]. Among individuals homozygous for C at this locus, all harbor PERK haplotype A or D in some combination. Individuals heterozygous (C/T) at this locus were heterozygous for haplotypes A, B, and/or D. Individuals homozygous for T at the GWAS risk locus were always homozygous for PERK haplotype B. This demonstrates that one of the two amino acid changes conferred by the B PERK haplotype that are not shared by the D haplotype may be responsible for the PSP risk evident on Chr. 2.

To confirm the relationship of LD amongst SNP alleles in PSP subjects, we genotyped 1,043 PSP cases for rs7571971, and the 3 coding variant SNPs. The genotypes for these four SNPs were then phased using maximum likelihood. We observed that, in PSP cases, haplotype frequencies were almost identical to those from 1000 Genomes data: for A, 0.645 versus 0.642; for B, 0.288 versus 0.301; and for D, 0.061 versus 0.053. Again haplotypes A and D are completely correlated with rs7571971 allele C (Figure 7), the protective PSP allele. Haplotype B is completely correlated with allele T, the high risk PSP allele. Recently Liu *et al.*[31] showed that when lymphoblastoid cell lines are treated with thapsigarin to induce ER stress, cells homozygous for the B haplotype showed stronger activation than cells homozygous for the A haplotype. Thus B is the high-risk haplotype for PSP suggesting that activation is not a protective response, but rather increases risk for PSP.

## Discussion

We found that PERK is activated in disease-affected brain regions in PSP, including the pons, medulla, and midbrain. We also found that PERK’s downstream effector, eIF2α, is activated similarly in PSP brainstem areas. In contrast, PERK and eIF2 are not activated or only weakly activated in normal and AD brainstem, respectively. We confirmed that AD cases have strong immunoreactivity for pPERK and peIF2α in the pyramidal cells of the hippocampus [25] and in the frontal cortex. In contrast, PSP cases show mild to moderate pPERK staining in these regions [26]. PERK and eIF2α were not activated in the cerebellum in either disease. In AD and PSP, the pattern of UPR activation pllels the regional distribution of pathology in these two disorders.

We explored the relationship between abnormal tau deposits and UPR activation in the PSP brainstem. Although there is some overlap between cells with activated PERK and cells with htau, at least half of htau-positive cells do not have concurrent PERK activation. A greater proportion of pPERK positive cells were also positive for tau, but 25% stained for pPERK alone. Thus, although PERK is activated in brain regions highly affected by tau pathology, htau and pPERK do not necessarily overlap at the single cell level. One potential explanation for lack of complete overlap may be that PERK activation precedes tangle formation, and is no longer activated in cells with mature tangles. We found that overlap between pPERK and htau mostly occurred in cells with diffuse htau staining rather than dense tau staining, supporting this hypothesis. Similarly, Hoozemans *et al.* (2009) found that cells in the AD hippocampus that were positive for pPERK also stained for markers of early tau aggregation [25]. This evidence suggests that PERK activation may temporally precede overt tau aggregation, and could be triggered by immunohistochemically undetectable levels of abnormal tau. The genetic data implicating both PERK and tau in PSP supports a plausible temporal relationship between PERK activation and tau aggregation.

Genetic findings [10] and the data presented here implicate PERK as well as the UPR in the pathogenesis of PSP. Genetic findings also associate *MAPT* with PSP [7,10,35,36], and along with the presence of aggregated tau as the key neuropathologic feature of PSP, these data clearly establish that tau is intimately linked to PSP pathogenesis. While the UPR is activated by misfolded proteins within the ER, and aggregated misfolded tau occurs in PSP, AD, and other tauopathies, tau is a cytosolic protein and does not appear to traffic through the ER as part of a secretory pathway. In normal neurons, most tau protein is intracellular and attached to microtubules. In tauopathies, tau aggregates in the cytoplasm of cells, in cellular processes, and at nerve terminals, but there is no evidence that tau aggregates in the ER. Recent work in mouse models of α-synucleinopathies [37,38] and studies on PD autopsy material [38] suggest that small amounts of α-synuclein can be found in ER, and that in the disease state, these levels are elevated, thereby activating the ER stress response. Still, since there is no direct evidence that tau traffics through the ER, or evidence of tau aggregates in the ER, it is unlikely that misfolded tau directly activates the canonical UPR. This view is supported by the fact that in PSP, pPERK and pEIF2α are activated in cells with no observable tau pathology, but we cannot exclude the possibility that very low or undetectable levels of aggregated tau are present. Rather, a more likely explanation is that tau-induced cytoplasmic events act to trigger the UPR by an unknown mechanism, which in turn influences the degradation of tau. A possible mechanism is that cytoplasmic aggregated tau may inhibit processes such as the ERAD-proteosome pathway used by cells to degrade misfolded ER proteins, and thus preventing the normal degradation of these proteins, stimulating ER stress [39].

PERK and eIF2α are also activated in pathology-associated regions of a number of other neurodegenerative diseases, including another tauopathy, AD [25]. The UPR is also activated in non-tau diseases that include ALS where UPR activation is observed in the spinal cord in sporadic cases [28], and in PD where UPR activation occurs in the substantia nigra [27]. Expanded-repeat huntingtin, the pathological protein in Huntington’s disease, induces ER stress in culture [40]. Notably, these diseases share a common pathology, *i.e.* the accumulation of abnormal aggregated proteins in the CNS. Thus, there may be a common mechanism by which aggregated cytoplasmic proteins activate the UPR. The genetic association between PERK and PSP suggests that this UPR activation can influence the disease process, at least in the case of PSP.

Surprisingly, we found that 10/14 normal controls over 50 years of age had at least minimal activation of pPERK in the hippocampus. This is in contrast to previous studies that report no pPERK staining in this region in normal controls [26]. In these subjects, the degree of pPERK immunoreactivity correlated positively with both the degree of htau immunoreactivity and age, but did not correlate with amyloid pathology. The presence of at least some tau pathology in the hippocampus of normal subjects is consistent with work by others [41], and could potentially indicate either pre-clinical AD or early neurofibrillary tangle predominant dementia (NFTD). However, in the absence of clinical symptoms, it is not possible to make either diagnosis. These findings in normal controls are consistent with the idea that the activation of the UPR is due to the tau pathology and not the amyloid pathology.

We reported strong genetic evidence that *EIF2AK3* genotypes confer risk for PSP [10]. The strongest signal comes from single nucleotide polymorphism (SNP) rs7571971 that is within *EIF2AK3*. There are several non-synonymous coding polymorphisms in *EIF2AK3* that track with risk and *EIF2AK3* appears to be the gene in this region involved in PSP. However, another less likely but still plausible explanation is that PSP risk in this region comes from a regulatory element that is intronic, within *EIF2AK3*, or in a close by intergenic region and that this element controls expression of another gene. Also, the true PSP association could be from nearby genes (e.g. *FOXI3* or *RPIA*) though this is less likely since the signal from SNPs in highlighting these genes are not as significant as SNPs within *EIF2AK3*. The work presented here clearly demonstrates that in PSP, PERK is activated in a region-specific pattern that matches regions where neurodegeneration occurs. Thus this functional evidence along with the strength of the genetic evidence indicates that *EIF2AK3* and not an adjacent locus is the gene that confers risk for PSP.

The SNP giving the strongest *EIF2AK3* signal in the PSP GWAS (rs7571971) is intragenic in intron 2. This SNP is in strong disequilibrium with 3 *EIF2AK3* exonic SNPs which are non-synonymous. This relationship was predicted using 1,000 Genomes data and confirmed here in PSP subjects (Figure 7). These coding variants form two common (A and B) and one rare haplotype (D). In PSP subjects, the low risk allele [C] at rs7571971 completely correlates with haplotypes A and D while the high risk rs7571971 allele [T] completely correlates with haplotype B (Figure 7).

Work in lymphoblastoid cell lines [31] with different haplotypes show that expression of EIF2AK3 is not altered by these haplotypes. However, when the PERK arm of the UPR is activated by thapsigargin, PERK from haplotype B homozygote cells is more active in phosphorylating eIF2α when compared to PERK from cells homozygous for haplotype A. The haplotype that confers high risk for PSP produces the more active form of PERK, suggesting that activation of the UPR is pathogenic in PSP and not a protective response. This is consistent with observations in prion protein induced neurodegeneration. Moreno *et al.*, showed that during prion replication, synaptic failure and neuronal loss is temporally associated with UPR activation and inhibition of translation. When translation is restored using over-expression of GADD34 to dephosphorylate eIF2α, survival of infected animals is prolonged. In contrast, when the UPR is activated using salubrinal, survival is decreased [42]. Both observations are consistent with activation of the PERK/eIF2α arm of the UPR enhancing neurodegeneration, as proposed here for PSP.

The two low risk haplotypes (haplotype A, Ser-Arg-Ser; and haplotype B, Ser-Gln-Ser) differ only at the middle amino acid, 166— this amino acid is unlikely to functionally influence PERK activation. The low and high risk (Haplotype B, Cys-Gln-Cys) haplotypes differ at both positions 166 and 704, and one or both may influence PERK activity. Amino acid 166 is on the portion of PERK that is in the ER lumen and positioned where this protein senses mis-folded proteins. Position 704 is on the cytoplasmic side of PERK, a segment of the protein that is phosphorylated when activated and that has the active site for phosphorylating eIF2α. Additional work is needed to confirm that haplotype B PERK is the more active protein and to determine if mis-folded protein sensing or activation *via* auto phosphorylation is affected.

## Conclusions

The PERK protein and its downstream effector eIF2α are phosphorylated in disease-affected regions in both PSP and Alzheimer’s disease. A previous study using PSP samples described UPR activation primarily in the hippocampus, a brain region not affected in this disease [26]. In contrast, we examined a large panel of brain areas (pons, medulla, midbrain, hippocampus, frontal cortex, and folia of the cerebellum) from PSP and AD cases as well as normal controls to show that this activation is disease-specific in its geographic distribution in the brain. In contrast to previous reports, we also found UPR activation in the hippocampus of a subset of our normal controls, a completely novel finding. This activation positively and significantly correlated with both age and amount of tau pathology. This suggests that tau and UPR activation are linked. We also demonstrated a genetic association between an EIF2AK3 protein coding haplotype and PSP, indicating that variation in the PERK protein affects PSP risk.

## Methods

### Human tissue

We obtained postmortem human pons, medulla, midbrain, frontal cortex, hippocampus, and cerebellum samples from the Center for Neurodegenerative Disease Research (CNDR; University of Pennsylvania School of Medicine, Philadelphia, PA) using the CNDR Integrated Neurodegenerative Disease Database [33] and from the Michigan Alzheimer’s Disease Research Center Brain Bank (MADRC; University of Michigan, Ann Arbor, MI). We chose PSP and AD cases for lack of co-morbid diagnoses and availability of fixed tissue-- PSP and AD cases with a secondary neuropathological diagnosis (NPDx; for instance, PD) were excluded from the present study. All PSP and AD cases were evaluated by a neurologist pre-mortem and referred to the CNDR or MADRC brain donation programs, where a neuropathologist made a NPDx according to established criteria [43,44]. Controls had no clinical history or postmortem diagnosis of a neurodegenerative disease. One control displayed a moderate amount of Lewy body pathology in the medulla and another displayed a mild amount of tau deposition in the midbrain (though not in the substantia nigra). All control cases were free of Lewy bodies in the hippocampus. We age-matched all cases and controls (See Table 4 and Additional file 1: Table S1 for demographic information). Tissue used for immunohistochemical and immunofluorescence studies was fixed in either ethanol (70%) or 10% neutral buffered formalin overnight and then processed for pffin embedding and 6 μm thick sections were generated as described [45] using a Leitz 1512 microtome. The average age of PSP, AD, and normal controls was approximately 75 years. Average disease duration for PSP patients was 6.7 years, while the average duration for AD patients was 10.8 years. The average post-mortem interval (PMI) for all cases was 10.2 hours (Table 4, and Additional file 1: Table S1). Pontine sections included the locus coeruleus and surrounding tegmentum, midbrain sections included the substantia nigra, medulla sections included the olivary nucleus, hippocampal sections included the CA and dentate regions, frontal cortex sections included both white and gray matter, and cerebellar sections included the folia.

**Table 4:** Subject information

	**n**	**Avg age of onset**	**Avg age at death**	**Avg disease duration**	**% female**	**Avg PMI**
**Normal – age-matched**	15	N/A	71.7(8.4)	N/A	46.7	12.7(8.5)
**Normal – non age-matched**	12	N/A	54.5(24.9)	N/A	66.7	9.1(3.4)
**Normal - total**	27	N/A	64.0(19.4)	N/A	56.7	11.1(6.9)
**PSP**	17	66.8(5.8)	73.7(5.2)	6. 7(2.2)	64.7	11.7(6.0)
**AD**	9	65.0(6.3)	75.8(5.1)	10. 8(3.4)	44.4	9.4(5.8)

There were no significant differences between groups for average age at death (ANOVA, p = 0.33), post-mortem interval (ANOVA, p = 0.54), average age of onset (PSP and AD only, Student’s *t*-test, p = .45) or percent female (Chi Square, p = 0.89). Average disease duration for AD was significantly longer than for PSP (Student’s *t*-test, p = 0.002). Standard deviations indicated inside parenthesis.

### Immunohistochemistry

Immunohistochemistry was performed as previously described [46,47]. We depffinized brain sections on slides using xylene (Mallinckrodt Baker Inc., Phillipsburg, NJ), and then hydrated them through a series of ethanol washes, and quenched endogenous peroxidases by immersing sections in a mixture of hydrogen peroxide and methanol. Following a wash in running water, we performed antigen retrieval by microwaving sections immersed in citrate buffer (Thermo Shandon Limited, Astmoor, WA). We then washed sections in 0.1 M Tris (pH 7.6; Fisher Scientific), blocked in 0.1 M Tris (pH 7.6)/2% fetal bovine serum (FBS), and applied primary antibody (incubated overnight at 4°). This wash/block procedure was identical for secondary antibody application, with an incubation time of one hr. Following another wash, we applied avidin/biotin complex (Vector Labs) to each section and incubated the sections for one hr. Finally, we developed sections with DAB chromagen (Biogenex), counterstained with hematoxylin, dehydrated through a series of ethanol and xylene washes. Cover slips were sealed with Cytoseal (Richard Allen Scientific, Kalamazoo, MI). Antibodies used are listed in Table 5.

**Table 5:** Anitbodies used

	**Antigen**	**Epitope**	**Source**	**Dilution**
**Primary**	pPERK	pThr981	Santa Cruz	1:4000
	peIF2α	pSer51	Sigma-Aldrich	1:2000
	AT8	pSer202/pThr205	Thermo Scientific	1:7500
**Secondary**	goat α rabbit IgG	biotin	Vector Labs	1:1000
	goat α rabbit IgG	AlexaFluor 488	Alexa	1:500
	goat α mouse IgG	AlexaFluor 594	Alexa	1:500

### Immunofluorescence (IF)

We depffinized, hydrated, quenched, and performed antigen retrieval on slide-mounted sections as described above. We then blocked sections in 0.1 M Tris/2% FBS, and applied mouse and rabbit primary antibodies (diluted in 0.1 M Tris/2% FBS). Primary antibody incubation time was 2 hr at room temperature. Following a wash in 0.1 M Tris and transfer to a “dark” chamber, we applied secondary antibodies (goat-anti-mouse and goat-anti-rabbit; Vector Labs) and let sections incubate for another two hr at room temperature. We then washed the sections again and applied 0.3% Sudan Black in 70% ethanol [48] for five min to quench endogenous lipofuscin related fluorophores. After another wash, the sections were coverslipped using Vectashield (with DAPI; Vector Labs; [49]).

### Slide scoring and analysis

pPERK and peIF2 antibodies both stained cells in a characteristic punctate pattern (Figures 1a and 2a; [25,27]). We scored each tissue section for pPERK or peIF2α IHC staining according to the following scale: *negative (−)*: no cells stained, *rare (R)*: 1–3 cells stained, *+*: 4–20 cells stained, *++*: 20+ cells stained, could have diffuse distribution of stained cells, may have high density of stained cells in some fields of the section, +++: high density of stained cells in almost every field of the section. A second rater confirmed scores in 20% of randomly selected slides (see Online Resource Figure 1). For double IF of hyperphosphorylated tau (htau) and pPERK, we visualized and photographed 10 fields per section and manually counted the number of htau positive cells, the number of pPERK positive cells, and the number of cells positive for both pPERK and htau. We scored all sections blind to disease group on an Olympus CHBS microscope (IHC) or an Olympus BX60 Transmitted-Reflected Light Microscope with BF/DF/DIC/Polarized Light and a SPOT RT Color digital microscope camera (IF).

### Statistical analysis

We used Spearman correlations to examine correlations between level of tau pathology vs. pPERK staining and age vs. pPERK staining, Fisher’s exact test to examine association between disease condition and pPERK/peIF2α staining, Chi Square to examine sex distribution among disease/normal groups, ANOVA to examine the mean difference among disease/normal groups for average age at death and post-mortem interval, and a Student’s *t*-test to examine the mean difference between disease groups for average age of onset. All statistical analyses were two-sided. Statistical significance was set at the 0.05 level unless otherwise indicated.

### Analysis of linkage disequilibrium around rs7571971

In a recent GWAS for PSP risk loci [10], a significant association was established between PSP risk and rs7571971. This SNP falls in an intron of *EIF2AK3*, the gene encoding PERK. While it is reasonable to assume the SNP somehow affects risk for PSP by affecting expression of *EIF2AK3*, it remains to be proven. To garner genetic evidence for this hypothesis, we first evaluated the pattern LD in sequence data from the 1000 Genomes project [50] and pairwise LD evaluated using SNAP (Suite of Nucleotide Analysis Programs, [51]). Based on these results, we genotyped 1043 PSP patients using TaqMan SNP Genotyping Assays (Applied Biosystems, Foster City, CA) for the following four SNPs: rs7571971, rs867529 (S136C), rs1805165 (S704A), and rs13045 (R166Q). All cases were autopsied and had a neuropathologic diagnosis of PSP [52]. Genotyping was done according to manufacturer’s protocol. PCR conditions were as follows: 95°C for 10 minutes, then 50 cycles of 92°C for 15 seconds, 60°C for 1 minute, 4°C for 2 minutes. Genotypes were visualized and called using a 7900HT Fast Real Time PCR System and the allelic discrimination function of Sequence Detection System V.2.4 (Applied Biosystems, Foster City, CA). Finally, we phased the resulting four-SNP genotypes using eHap software [53], which provides maximum likelihood estimates of haplotype frequencies. Approval for the use of de-identified patient samples was approved by the University of Pennsylvania Institutional Review Board (IRB); sample and demographic information collection at each participating site was approved by institution-specific IRB. Informed consent was obtained for all samples collected in a clinical setting.

## Competing interests

The authors declare that they have no competing interests.

## Authors’ contributions

LS carried out the immunostaining experiments and scoring, performed the Taqman genotyping, participated in the genotyping analysis, and drafted the manuscript. SX performed statistical analysis of immunostaining experiments. AN performed statistical analysis on genotyping data. RA and SG characterized, prepared and contributed a subset of tissue samples from patients with PSP. PSP-GSG characterized, prepared and contributed genetic samples from patients with PSP. VMYL and JQT contributed to study design and characterized, prepared and contributed tissue samples from PSP, AD and normal controls. BD performed linkage disequilibrium analysis and participated in drafting the manuscript. GDS conceived of the study, contributed to study design and coordination, and helped to draft the manuscript. All authors read and approved the final manuscript.

## Supplementary Material

Additional file 1: Figure S1 Scoring system examples. Representative fields from brain areas that scored “-“ (negative), “R” (rare), “+” (mild staining), “++” (moderate staining), and “+++” (heavy staining), along with scoring criteria. **Table S1.** Individual Case Information. Click here for file

## PSP Genetics Study Group information

Günter U. Höglinger^49^, Ulrich Müller^50^, Dennis W. Dickson^8^, Rosa Rademakers^8^, Rohan de Silva^9^, Irene Litvan^10^, Rachel G Gross^29^, John Hardy^9^, Peter Heutink^42^, Howard I Hurtig^29^, Matthew J Farrer^43^, Ryan J Uitti^12^, Jana Vandrovcova^9^, John C. van Swieten^11^, Zbignew K. Wszolek^12^, Eduardo Tolosa^13^, Pau Pastor^14-16^, Tamas Revesz^17^, Andrew J. Lees^9,17^, Elena Alonso^14,15^, Manuela Apfelbacher^18^, Steven E. Arnold^44^, Jesus Avila^19^, Thomas G. Beach^20^, Agnita J.W. Boon^11^, Wang Zheng Chiu^11^, Laura Donker Kaat^11^, Peter P. De Deyn^45^, Ranjan Duara^46^, Sebastiaan Engelborghs^45^, Yvette Bordelon^21^, Justo García de Yebenes^22^, NiCole A. Finch^8^, Owen A. Ross^8^, Robyn Flook^23^, Matthew P. Frosch^24^, Carles Gaig^13^, Douglas R. Galasko^25^, Eliezer Masliah^25,26^, Marla Gearing^27^, Bernadino Ghetti^28^, Salvatore Spina^28^, Neill R. Graff-Radford^12^, Murray Grossman^29^, Jorge L. Juncos^30^, Hans A. Kretzschmar^31^, Andrew P. Leiberman^32^, Kelly E Lyons^47^, Luke A. Massey^9^, Sean S. O’Sullivan^9^, Rajesh Pahwa^47^, Laura Silveira-Moriyama^9^, Catriona A. McLean^33^, Alberto Rabano^34^, Alex Rajput^35^, Sigrun Roeber^31^, Jonathan D. Rohrer^36^, Martin N. Rossor^36^, Wallace W Tourtellotte^48^, Claire Troakes^37^, Safa Al-Sarraj^37^, Juan C. Troncoso^38^, Vivianna M. Van Deerlin^1,2^, Jean Paul G. Vonsattel^39^, Charles L. White^40^ and Chris Zarrow^41^.

^8^Department of Neuroscience, Mayo Clinic, Jacksonville, Florida, USA. ^9^Reta Lila Weston Institute, University College London (UCL) Institute of Neurology, London, UK. ^10^Department of Neurology, Division of Movement Disorders, University of Louisville, Louisville, Kentucky, USA. ^11^Department of Neurology, Erasmus University Medical Center, Rotterdam, The Netherlands. ^12^Department of Neurology, Mayo Clinic, Jacksonville, Florida, USA. ^13^Neurology Service, Centro de Investigación Biomédica en Red sobre Enfermedades Neurodegenerativas (CIBERNED), Hospital Clínic, Institut d’Investigacions Biomèdiques August Pi i Sunyer (IDIBAPS), Universitat de Barcelona, Barcelona, Spain. ^14^CIBERNED, Instituto de Salud Carlos III, Madrid, Spain. ^15^Neurogenetics laboratory, Division of Neurosciences, University of Navarra Center for Applied Medical Research, Pamplona, Spain. ^16^Department of Neurology, University of Navarra, Clínica Universidad de Navarra, Pamplona, Spain. ^17^Department of Molecular Neuroscience, Queen Square Brain Bank for Neurological Disorders, UCL Institute of Neurology, University College London, London, UK. ^18^Institute of Legal Medicine, University of Würzburg, Würzburg, Germany. ^19^Centro de Biologia Molecular Severo Ochoa (CSIC-UAM), Campus Cantoblanco, Universidad Autonoma de Madrid, Madrid, Spain. ^20^Civin Laboratory for Neuropathology, Banner Sun Health Research Institute, Sun City, Arizona, USA. ^21^Department of Neurology, University of California Los Angeles, Los Angeles, California, USA. ^22^Department of Neurology, Hospital Ramón y Cajal, Madrid, Spain. ^23^Centre for Neuroscience, Flinders University and Australian Brain Bank Network, Victoria, Australia. ^24^C.S. Kubik Laboratory for Neuropathology, Massachusetts General Hospital and Harvard Medical School, Boston, Massachusetts, USA. ^25^Department of Neurosciences, University of California San Diego, La Jolla, California, USA. ^26^Department of Pathology, University of California San Diego, La Jolla, California, USA. ^27^Department of Pathology and Laboratory Medicine, Emory University School of Medicine, Atlanta, Georgia, USA. ^28^Department of Pathology and Laboratory Medicine, Indiana University School of Medicine, Indianapolis, Indiana, USA. ^29^Department of Neurology, University of Pennsylvania Health System, Philadelphia, Pennsylvania, USA. ^30^Department of Neurology, Emory University, Atlanta, Georgia, USA. ^31^Institut für Neuropathologie, Ludwig-Maximilians-Universität and Brain Net Germany, Munich, Germany. ^32^Department of Pathology, University of Michigan Medical School, Ann Arbor, Michigan, USA. ^33^Victorian Brain Bank Network, Mental Health Research Institute, Victoria, Australia. ^34^Department of Neuropathology and Tissue Bank, Fundación Centro Investigación Enfermedades Neurológicas (CIEN), Instituto de Salud Carlos III, Madrid, Spain. ^35^Division of Neurology, Royal University Hospital, University of Saskatchewan, Saskatchewan, Canada. ^36^Department of Neurodegenerative Disease, Dementia Research Centre, UCL Institute of Neurology, UCL, London, UK. ^37^MRC London Neurodegenerative Diseases Brain Bank, Department of Clinical Neuroscience, Institute of Psychiatry, King’s College London, London, UK. ^38^Department of Neurology, Johns Hopkins University School of Medicine, Baltimore, Maryland, USA. ^39^Department of Pathology and the Taub Institute for Research on Alzheimer’s Disease and the Aging Brain, Columbia University, New York, New York, USA. ^40^Department of Pathology, University of Texas Southwestern Medical Center, Dallas, Texas, USA. ^41^Rancho Los Amigos National Rehabilitation Center, University of Southern California, Downey, California, USA. ^42^Department of Clinical Genetics, Vrije Universiteit (VU) Medical Center, Section Medical Genomics, Amsterdam, The Netherlands. ^43^Department of Medical Genetics, University of British Columbia, Vancouver, British Columbia, Canada. ^44^Department of Psychiatry, Center for Neurobiology and Behavior, University of Pennsylvania School of Medicine, Philadelphia, Pennsylvania, USA. ^45^Department of Biomedical Sciences, University of Antwerp, Antwerp, Belgium. ^46^Wien Center for Alzheimer’s Disease and Memory Disorders, Mt. Sinai Medical Center, Miami Beach, Florida, USA. ^47^Department of Neurology, University of Kansas Medical Center, Kansas City, Kansas, USA. ^48^Human Brain and Spinal Fluid Resource Center, Veterans Affairs West Los Angeles Healthcare Center, Los Angeles, California, USA. ^49^Department of Neurology, Philipps-Universität, Marburg, Germany. ^50^Institut for Humangenetik, Justus-Liebig-Universität, Giessen, Germany.
