# Supplementary material for: The unfolded protein response is activated in disease-affected brain regions in progressive supranuclear palsy and Alzheimer’s disease
Source: Acta Neuropathol Commun. 2013 Jul 6;1:31. doi: 10.1186/2051-5960-1-31 (PMC3893579; doi:10.1186/2051-5960-1-31)
Supplement: Additional file 1: Figure S1 — Scoring system examples. Representative fields from brain areas that scored “-“ (negative), “R” (rare), “+” (mild staining), “++” (moderate staining), and “+++” (heavy staining), along with scoring criteria. Table S1. Individual Case Information. [file 2051-5960-1-31-S1.docx]

**Supplementary Material**

**Figure S1.** Scoring system examples. Representative fields from brain areas that scored “-“ (negative), “R” (rare), “+” (mild staining), “++” (moderate staining), and “+++” (heavy staining), along with scoring criteria.


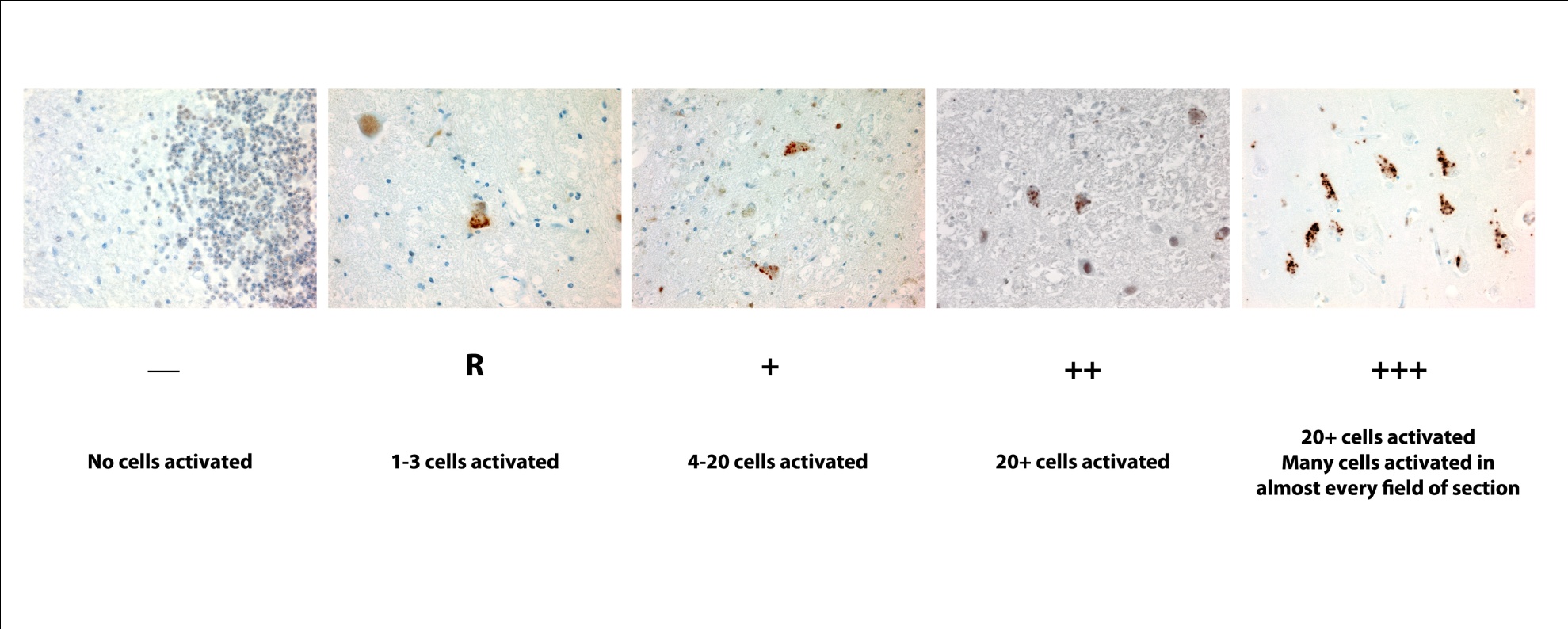


**Table S1.** Individual Case Information.

| **Case Demographics** | | | | | | |
| --- | --- | --- | --- | --- | --- | --- |
| **Case #** | **NPDx** | **Sex** | **Age at Death** | **Disease Duration** | **Post-Mortem Interval** | ***EIF2AK3* haplotype** |
| 1 | PSP | M | 73 | 6 | 20.5 | A/B |
| 2 | PSP | F | 70 | 7 | 4.5 | A/A |
| 3 | PSP | F | 79 | 11 | 17 | B/B |
| 4 | PSP | M | 73 | 4 | 10.5 |  |
| 5 | PSP | F | 72 | 5 | 20 |  |
| 6 | PSP | F | 71 | 6 | 6.5 | A/A |
| 7 | PSP | F | 81 | 7 | 17 | A/A |
| 8 | PSP | F | 77 | 11 | 12.5 | A/B |
| 9 | PSP | M | 85 | 3 | - |  |
| 10 | PSP | F | 75 | 5 | 12 | B/B |
| 11 | PSP | F | 76 | 8 | 8 | A/A |
| 12 | PSP | M | 67 | 10 | 12 | A/A |
| 13 | PSP | M | 70 | 6 | 11 | B/B |
| 14 | PSP | F | 78 | 8 | 8 | A/D |
| 15 | PSP | F | 64 | 7 | 21 | A/B |
| 16 | PSP | M | 72 | 7 | 3.5 | A/A |
| 17 | PSP | F | 60 | 3 | 3 | B/D |
| 18 | Normal | M | 60 | - | 14 | A/B |
| 19 | Normal | F | 75 | - | 3.5 | A/D |
| 20 | Normal | F | 70 | - | 10.5 | A/A |
| 21 | Normal | M | 67 | - | 10.5 |  |
| 22 | Normal | M | 90 | - | 6 | A/B |
| 23 | Normal | M | 74 | - | 7.5 |  |
| 24 | Normal | F | 83 | - | 3 | A/B |
| 25 | Normal | F | 75 | - | 15 |  |
| 26 | Normal | M | 83 | - | 14.5 | A/A |
| * 27 | Normal | M | 63 |  | 5 |  |
| * 28 | Normal | M | 70 |  | 19 |  |
| * 29 | Normal | F | 67 |  | 5.5 |  |
| †* 30 | Normal | M | 65 | - | 26 |  |
| † 31 | Normal | F | 89 | - | 7 |  |
| † 32 | Normal | F | 72 | - | 4 |  |
| † 33 | Normal | F | 51 | - | 5 |  |
| † 34 | Normal | F | 92 | - | 5 |  |
| † 35 | Normal | F | 68 | - | 32 |  |
| † 36 | Normal | F | 85 | - | 14 |  |
| †* 37 | Normal | F | 65 | - | 19 | A/B |
| † 38 | Normal | F | 56 | - | 12 |  |
| † 39 | Normal | F | 46 | - | 12 |  |
| † 40 | Normal | M | 47 | - | 11 |  |
| † 41 | Normal | M | 38 | - | 12 |  |
| † 42 | Normal | M | 16 | - | 8 |  |
| † 43 | Normal | F | 33 | - | 7 |  |
| † 44 | Normal | M | 29 | - | 12 |  |
| 45 | AD | F | 71 | 12 | 17 |  |
| 46 | AD | M | 85 | 13 | 6.5 |  |
| 47 | AD | F | 72 | 17 | 12 | A/A |
| 48 | AD | F | 81 | 11 | 4 | A/B |
| 49 | AD | M | 70 | 10 | 5 | A/A |
| 50 | AD | M | 77 | 6 | 4 | B/B |
| 51 | AD | M | 74 | 12 | 7.5 | A/B |
| 52 | AD | M | 73 | 10 | 9 | A/B |
| 53 | AD | F | 79 | 6 | 20 | A/A |

Neuropathological diagnosis, sex, age at death, disease duration, post-mortem interval, Braak stage, and hippocampal Aβ amyloid plaque score for all cases and controls used

† = hippocampus only

* = pons, cerebellum and/or hippocampus only.
